# 25-Hydroxycholesterol protects against myocardial ischemia-reperfusion injury via inhibiting PARP activity

**DOI:** 10.7150/ijbs.35075

**Published:** 2020-01-01

**Authors:** Suying Lv, Chenhui Ju, Jiangtong Peng, Minglu Liang, Feng Zhu, Cheng Wang, Kai Huang, Min Cheng, Fengxiao Zhang

**Affiliations:** 1 Department of Cardiology, Union Hospital, Tongji Medical College, Huazhong University of Science and Technology, Wuhan, China; 2 Clinic Center of Human Gene Research, Union Hospital, Tongji Medical College, Huazhong, University of Science and Technology, Wuhan, China; 3 Department of Rheumatology, Union Hospital, Tongji Medical College, Huazhong University of Science and Technology, Wuhan, China

**Keywords:** myocardial ischemia-reperfusion, myocardial apoptosis, 25-hydroxycholesterol, PARP

## Abstract

Myocardial ischemia-reperfusion (IR) injury occurs when occlusive coronary artery restores blood supply after events such as myocardial infarction, stroke, cardiac arrest and resuscitation, and organ transplantation. However, the mechanisms involved are poorly understood, and effective pharmacological interventions are still lacking. A previous study demonstrated that 25-hydroxycholesterol (25-HC) contributed to lipid metabolism and cholesterol metabolism as an oxysterol molecule. We herein explored whether 25-hydroxycholesterol (25-HC) has cardioprotective properties against IR injury and explored its underlying mechanisms. 25-HC was administered before reperfusion procedure in IR injury model mice. We found that 25-HC significantly reduced the IR-induced infarct size and improved cardiac function, and this protective effect was associated with reduced phosphorylation of p38-MAPK and JNK1/2. Besides, 25-HC also inhibited the Bax/Bcl-2 ratio and the relative expression of cleaved caspase-3. Furthermore, 25-HC decreased the PARP activity, indicating that 25-HC ameliorates IR injury via the PARP pathway. The 25-HC group abolished cardioprotection in the presence of little PARP activity, suggesting that the PARP activity is essential for 25-HC to exert its effect during IR injury. Our primary study indicates that 25-HC ameliorated IR injury by inhibiting the PARP activity and decreasing myocardial apoptosis, which makes it a potential therapeutic drug in IR injury of the heart.

## Introduction

Myocardial ischemia-reperfusion (IR) injury is considered as a pathophysiological process wherein the ischemic tissue suffers from undesirable outcomes when occlusive coronary artery restores blood flow and oxygen and nutrients are subsequently supplied after several clinical events, such as myocardial infarction, stroke, cardiac arrest and resuscitation, and organ transplantation 1, 2. Accumulating evidence shows that myocardial apoptosis is a vital detrimental factor during IR injury that leads to reduced number of alive and beating myocytes and decreased heart function 3, 4. However, effective therapeutic targets and drugs for myocardial IR injury remain largely unknown.

During the process of IR injury, myocytes experience high levels of oxidative stress, which leads to DNA strand breaks; this subsequently triggers strong poly(ADP-ribose) polymerase (PARP) activity, thus hindering cell survival 5. Through poly(ADP-ribosyl)ation of target proteins, activated PARPs could modulate various beneficial cellular physiological processes, including transcriptional regulation, DNA repair, and duplicate regulation 6. However, the PARP hyperactivation results in cellular damage and cell death by necrosis and apoptosis in the presence of IR injury and hydrogen peroxide (H_2_O_2_) stimulation 7, 8. In addition, PARP1 knockout (PKO) which inhibited more than 90% PARP activity of the mice, has been reported to be protective in reperfused hearts 9, 10.

In living organisms, 25-hydroxycholesterol (25-HC) is mainly synthesized by cholesterol 25-hydroxylase (Ch25H) 11. Previous studies demonstrated that the biological activity of 25-HC is exerted via a ligand of liver X receptors (LXRs). The biological roles are in a feedback regulating system for cholesterol and bile acid metabolism, fatty acid and glucose metabolism, and lipid homeostasis and cancer therapeutics^12^-^14^. Furthermore, 25-HC maintains mitochondrial integrity and prevents the activation of inflammatory responses by modulating the DNA sensor protein that is absent in melanoma 2 (AIM2) 15. Interestingly, 25-HC exerts pro-inflammatory and anti-inflammatory activity depending on the complex cellular and immunological backgrounds 16, 17. Previous studies also reported that 25-HC affects sterol biosynthesis and cholesterol homeostasis 18, 19. However, patients with disrupted expression of the 25-HC-related gene display highly elevated (100-fold) levels of 25-HC but normal levels of cholesterol and bile acids 20. Despite varying results from numerous similar studies, the effects of 25-HC in the context of IR injury remain unclear. Therefore, our study aimed to clarify the role of 25-HC in myocardial IR injury and the potential mechanisms involved in the process.

## Materials and Methods

### Animals and IR surgery

Eight 2-week-old male C57BL/6 mice were bought from Charles River Laboratories China (Beijing, China). PARP1 knockout mice (PARP1^-/-^, 129S-Parp1tm1Zqw/J, PKO) were purchased from the Jackson Laboratory (Bar Harbor, Maine, USA) and were converted to the C57BL/6 background by reproducing more than ten generations. Eight-to-ten- week-old PKO mice having the C57BL/6 background were used for further experiments. All mice were housed in agreement with the guidelines of the Guide for the Care and Use of Laboratory Animals, and the experimental procedures implemented were approved by the Institutional Authority for Laboratory Animal Care of China. Briefly, each mouse was anesthetized using the intraperitoneal administration of sodium pentobarbital (50 mg/kg, Sigma, USA) in physiological saline. Subsequently, the anesthetized mouse was ventilated through endotracheal intubation for assisting respiration. The hearts were subjected to myocardial ischemia by ligating the left anterior descending (LAD) artery at a level approximately 1 mm below the edge of the left auricular appendix under a surgical microscope. A 7-0 silk suture required to be tied in a slipknot with a section of silica gel tubing. After 30 min, the knot was opened, and myocardial reperfusion was visually confirmed under a surgical microscope. Mice (n = 45) were randomly allocated into three groups: control group, sham group, and 25-HC group. In sham-operated animals, the same procedure was performed without LAD ligation. The sham group was subjected to the same operation without occlusion; the 25-HC group was subjected to IR surgery and intraperitoneal administration of 25-HC (10 mg/kg, Sigma, USA); and the control group was subjected to IR surgery and was administered equivalent volumes of 1:1 saline and ethanol mixture.

### Echocardiography assessment

Briefly, before experiment and after 24 h of reperfusion, mice were lightly anesthetized by isoflurane inhalation (Sigma, USA) in O_2_ gas and placed in a supine position on a 37° constant- temperature heating pad. Echocardiography was performed using an echocardiographic imaging system (Vevo 770, VisualSonic, Canada). The ultrasound probe was applied to the left mid‐ventricular level to find the papillary muscles, and 2D-targeted M-mode images were recorded. Cardiac function was assessed using left ventricular ejection fraction (LVEF) and left ventricular fractional shortening (LVFS), which were calculated as the respective average values for five consecutive cardiac cycles. Cardiac function was detected and analyzed by an observer blinded to treatment.

### Measurement of myocardial infarct size

After 24 h of reperfusion, the hearts were treated with Evans blue/triphenyl tetrazolium chloride (TTC) staining. In brief, the ligature around the LAD coronary artery was retied, and the ascending aorta was injected with approximately 0.1 mL of 2% Evans blue dye. Subsequently, the heart was quickly removed, cleaned, frozen at -80°C in a refrigerator (Thermo scientific, USA) for 10 min. Then, it was sliced into six 1-mm slices and incubated in 1% TTC (Sigma, USA) solution at 37°C for 15 min to demarcate the infarcted myocardium (I). The size of the area at risk (AAR) , which was represented as the unstained Evans blue region of the myocardium, was presented as the ratio of AAR over the total left ventricle (LV). Myocardial infarct size, which was represented as unstained TCC region of the myocardium, was presented as the ratio of I over AAR. The data were calculated using the computer graphics software Image-Pro Plus.

### Culturing of primary cardiomyocytes

Neonatal murine ventricular cardiomyocytes from 1- to 3-day-old C57BL/6 mice (Laboratory Animal Center, Huazhong University of Science and Technology) were isolated by digestion with type II collagenase (Worthington, USA) and cultured as previously described.

### TUNEL staining

To investigate apoptosis, TUNEL staining of heart tissues and primary cardiomyocytes was performed according to the protocol of TUNEL Staining Kit (Roche, USA). Cell nuclei labeled with 4,5-diamino-2-phenylindole (DAPI) appeared in blue color, and apoptotic nuclei labeled with TUNEL- positive staining were detected by red fluorescent staining.

### Western blot assay

Cells or heart tissues were lysed and incubated for 30 min in RIPA buffer supplemented with a proteinase inhibitor cocktail (Thermo Scientific, USA) and a phosphatase inhibitor (Thermo Scientific, USA). Protein concentration of the lysate was measured using the BCA protein assay kit (Thermo Scientific, USA). Equal amounts of protein (30-40 μg/lane) were submitted to 9% or 12% SDS polyacrylamide gels, depending on the target proteins. Then, they were transferred on polyvinylidene fluoride membranes (Millipore, USA), followed by overnight incubation with the following primary antibodies: P38 antibody (1:1000, Proteintech), JNK antibody (1:1000, Proteintech), ERK1/2 antibody (1:1000, Proteintech), phospho-p38 MAPK antibody (1:1000, CST), phospho-SAPK/JNK antibody (1:1000, CST), phospho-p44/p42 MAPK (Erk1/2) antibody (1:1000, CST), Bax antibody (1:1000, CST), BCL2 antibody (1:1000, CST), caspase-3 antibody (1:1000, Proteintech), cleaved caspase-3 antibody (1:1000, CST), poly(ADP-ribose) polymerase1 (parp1) antibody (1:1000, CST), GAPDH antibody (1:10000, Abcam), and anti-poly(ADP-ribose) polymer (PAR) antibody (1:1000, CST). Subsequently, membranes were incubated with corresponding secondary antibody, and specific protein bands were detected using the Bio-Rad imaging system (Hercules, CA).

### *In vitro* poly(ADP-ribosyl)ation assay

Nuclear extracts from primary mice cardiomyocytes were incubated with NAD^+^ and activated DNA in poly(ADPribosyl)ation assay buffer (Trevigen, USA) or with Pj34 (10 mM, Sigma, USA) for 30 min at 37°C. Poly(ADP-ribosyl)ation of nuclear extracts was then subjected to Western blot.

### RNA extraction and qRT-PCR

Total RNA was extracted from primary cardiomyocytes or heart tissues with TRIzol reagent (Takara, Japan). RNA was then reverse-transcribed using PrimeScript RT Reagent Kit (Takara, Japan). Real-time qRT-PCR was performed using TB Green Premix Ex Taq II Kit (Takara, Japan) in StepOne-Plus Real-Time PCR System (Thermo Scientific, USA). The relative expression of genes normalized to the endogenous control was calculated using the comparative Ct method formula 2^-ΔΔCt^. GAPDH was used as control. The real-time PCR primer sequences are shown in Table 1.

### The cell counting kit-8 (CCK8) assay

The measurement of cell vitality was performed using CCK8 kit (Dojindo, Japan) according to the manufacturer's protocol. Cells were incubated beforehand using the different concentration of 25-HC gradient. Cells were then stimulated with H_2_O_2_ (300 μM, Sigma, USA). After H_2_O_2_ stimulation for 12 h, the CCK8 solution was added to 96-well culture plates in the dark condition. After 2 h of incubation, absorbance (OD) was first read at 450 nm. Relative cell vitality was calculated according to the following formula: (OD test - OD blank)/(OD control - OD blank).

### Statistical analyses

Values were shown as means ± SEM of at least three independent experiments. Student's *t*-test was used to estimate the statistical difference between two groups. One-way ANOVA with Bonferroni test was used to evaluate variables among groups. GraphPad Prism 5.0 software (GraphPad Software Inc., La Jolla, CA) was used for statistical analyses. Statistical significance was defined as *p* < 0.05.

## Results

### Protection by 25-HC against ischemia-reperfusion injury in IR hearts

**Figure 1:**
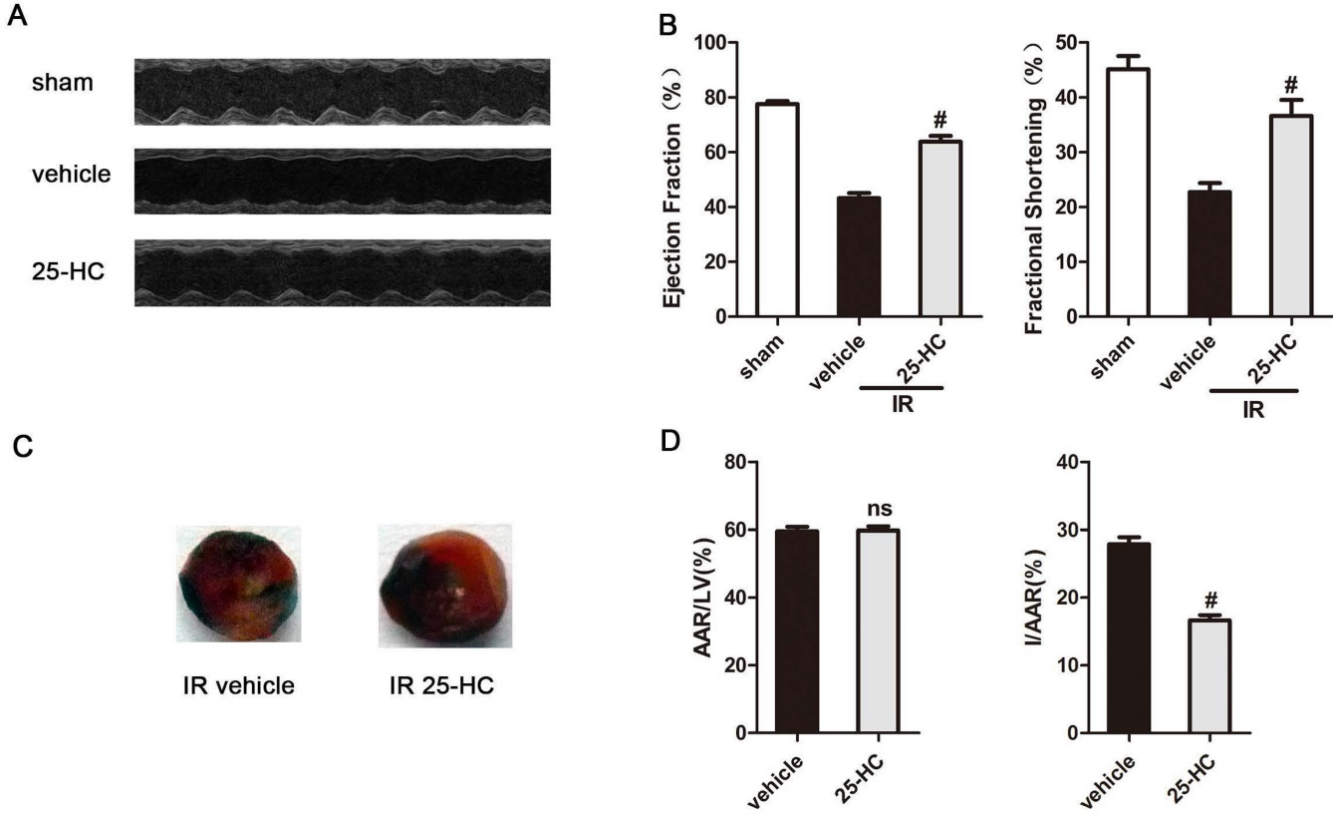
25-HC protects against IR injury. (A) Representative M-mode images and (B) statistical results (ejection fraction and fractional shorting) showed that cardiac systole dysfunction in IR mice was ameliorated by 25-HC pretreatment (10 mg/kg). (C) Representative photographs of Evans blue/TTC double-stained murine heart slices obtained 24 h after IR injury. Blue, remote area; white, infarct area; red and white, AAR. (D) Graphical representation of the LV infarct size and AAR. Infarct size relative to AAR (I/AAR), and AAR relative to left ventricle (AAR/LV). Mean ± SEM. N = 5-6 per group, *P < 0.05 vs. vehicle group, #P < 0.01 vs. vehicle group.

To investigate if 25-HC is involved in heart IR injury in mice, it was administered intraperitoneally (10 mg/kg) 10 min before reperfusion procedure in IR injury model mice. These mice had already undergone 30 min of ischemia and 24 h of reperfusion of LAD. There were no significant differences in weight loss or survival among groups (data no shown). Echocardiography measurements showed that mice undergoing IR injury (the vehicle group) presented significantly reduced LVEF% and LVFS% compared with the sham group (EF: 45.70% ± 0.62% vs. 77.34% ± 0.68%, P < 0.01; FS: 22.36% ± 0.45% vs. 45.70% ± 45.70%, P < 0.01; Figures 1A and 1B). Interestingly, 25-HC treatment (the 25-HC group) showed improved LVEF% and LVFS% compared with the vehicle group (EF: 66.13% ± 1.03% vs. 45.70% ± 0.62%, P < 0.01; FS: 36.61% ± 0.96% vs. 22.36% ± 0.45%, P < 0.01; Figures 1A and 1B). In addition, we set up three concentrations within the 25-HC group (5 mg/kg, 10 mg/kg, and 20 mg/kg) and found that 10-mg/kg 25-HC showed the most evident increase in the cardiac function parameters LVEF and LVFS (Figures S1A and S1B). Evans blue/TTC double staining was used to detect myocardial infarct size. Results showed that the infarct area was also significantly reduced in the 25-HC group than in the vehicle group (I/AAR: 27.88% ± 0.41% vs. 16.64% ± 0.31%, P < 0.01; Figures 1C and 1D). The data suggested a protective role of 25-HC in IR injury in ischemic heart disease.

### 25-HC suppresses IR-induced cardiac apoptosis

**Figure 2:**
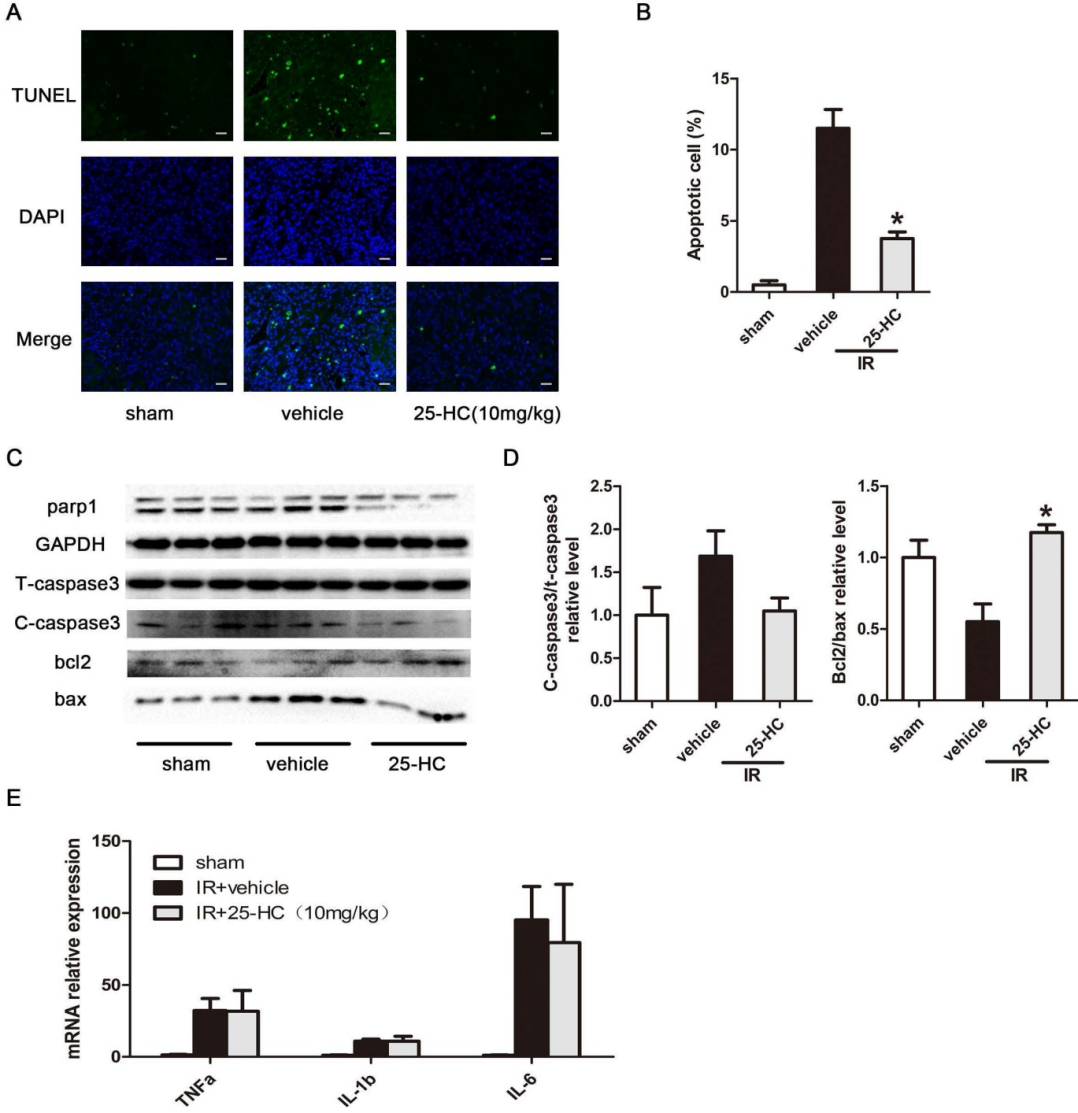
25-HC suppresses myocardial apoptosis in IR hearts. (A) Representative images of TUNEL staining showing cardiac cell apoptosis. (B) The percentage of apoptotic cells was shown. (C) The protein levels of T-caspase-3, Bax, Bcl-2, and PARP1 in left ventricular tissues. (D) Statistical results were shown. (E) The mRNA levels of TNF-α, IL-6, and IL-1β in left ventricular tissues. Mean ± SEM. N = 5-6 per group, *P < 0.05 vs. vehicle group.

Myocardial apoptosis plays a critical role in IR-induced cardiac damage and contributes greatly to impaired cardiac function 3, 21. The viability of cardiomyocytes was measured by the TUNEL assay. Results revealed that IR injury significantly promoted myocardial apoptosis in the infracted apical region, and this effect was inhibited by 25-HC treatment (Figure 2A and 2B). Furthermore, IR injury induced increased pro-apoptotic protein expression of cleaved caspase-3 and decreased anti-apoptotic protein expression of Bcl-2; conversely, 25-HC administration reversed these changes (Figure 2C and 2D). The mRNA expression of inflammatory cytokines, including TNF-α, IL-6, and IL-1β, were dramatically increased after IR injury. However, the inhibition of these inflammatory cytokines was not observed in the 25-HC group (Figure 2E), indicating that 25-HC medicated cardioprotective role mainly by anti-apoptosis but not by anti-inflammation.

### 25-HC inhibited ischemic injury by mediating PARP activity

**Figure 3:**
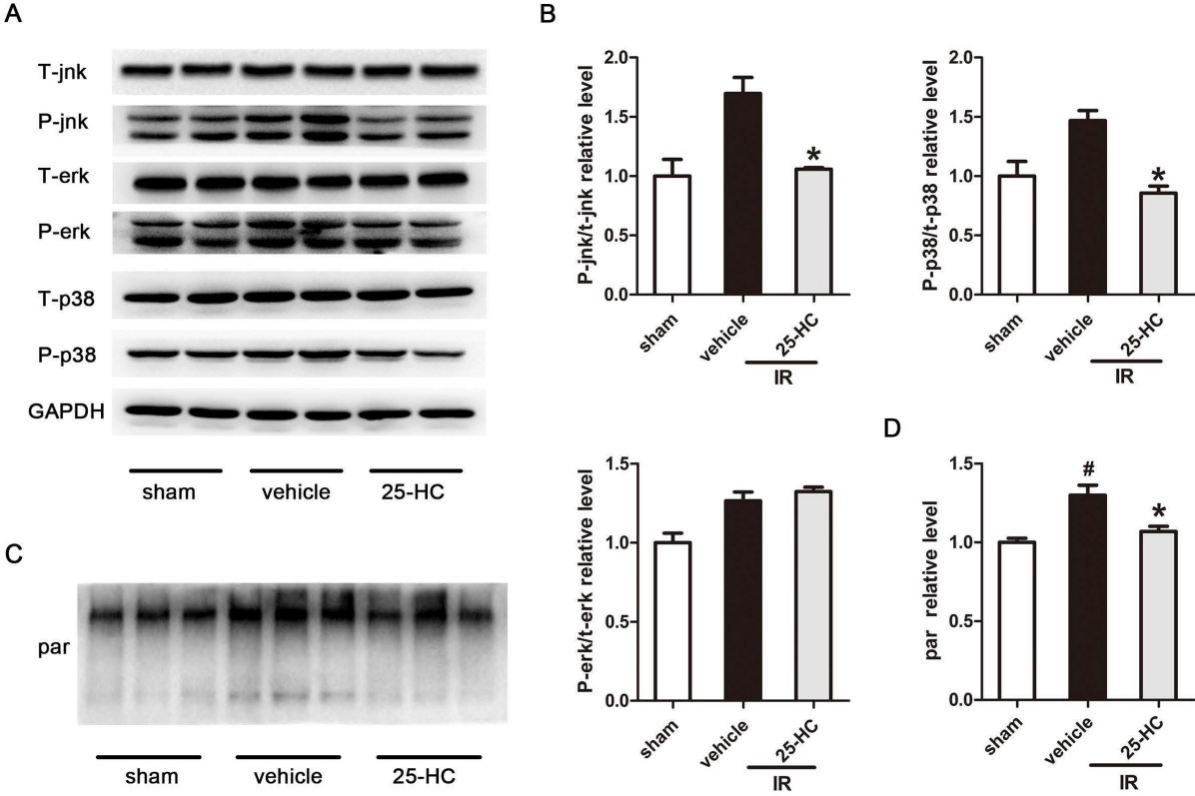
The effect of 25-HC on AMPK signaling. (A) C57/B6 mice were intraperitoneally injected with 25-HC in IR models, and the left ventricular tissues were subjected to Western blot assay with anti-p38, JNK, and ERK antibodies. (B) Representative statistical results in each protein. (C) C57/B6 mice were intraperitoneally injected with 25-HC in IR models; the left ventricular tissues were subjected to Western blot assay with anti-PAR antibody. (D) The statistical relative expression level of poly(ADP-ribosyl)ated protein. Mean ± SEM. N = 5-6 per group. *P < 0.05 vs. vehicle group, #P < 0.01 vs. sham group.

Classic factors of mitogen-activated protein kinase (MAPK), including ERK, p38-MAPK, and JNK, are the pivotal signal transducers involved in ischemia-induced apoptosis and cardiac dysfunction 22, 23. MAPK signaling was evaluated to examine the potential mechanisms driving the cardioprotective effect of 25-HC against MI and IR. Our data revealed that IR markedly augmented the phosphorylation of JNK and p38-MAPK, which was markedly attenuated by 25-HC (Figure 3A, and 3B). However, ERK phosphorylation did not significantly differ between the vehicle group and the 25-HC group. These data proved that 25-HC prevented myocardial apoptosis by reducing the phosphorylation of JNK and p38-MAPK. A previous study proved that PARP activation was closely associated with MAPK signal activation 24, 25. Therefore, we detected the PARP activity of the myocardium in IR mice. The poly(ADP-ribosyl)ated proteins were tested by Western blot assay with anti-poly(ADP-ribose) polymer (PAR) antibody. The results showed that myocardial reperfusion resulted in dramatically increased the PARP activity, while the increased PARP activity was reversed by 25-HC treatment (Figure 3C and 3D).

**Figure 4:**
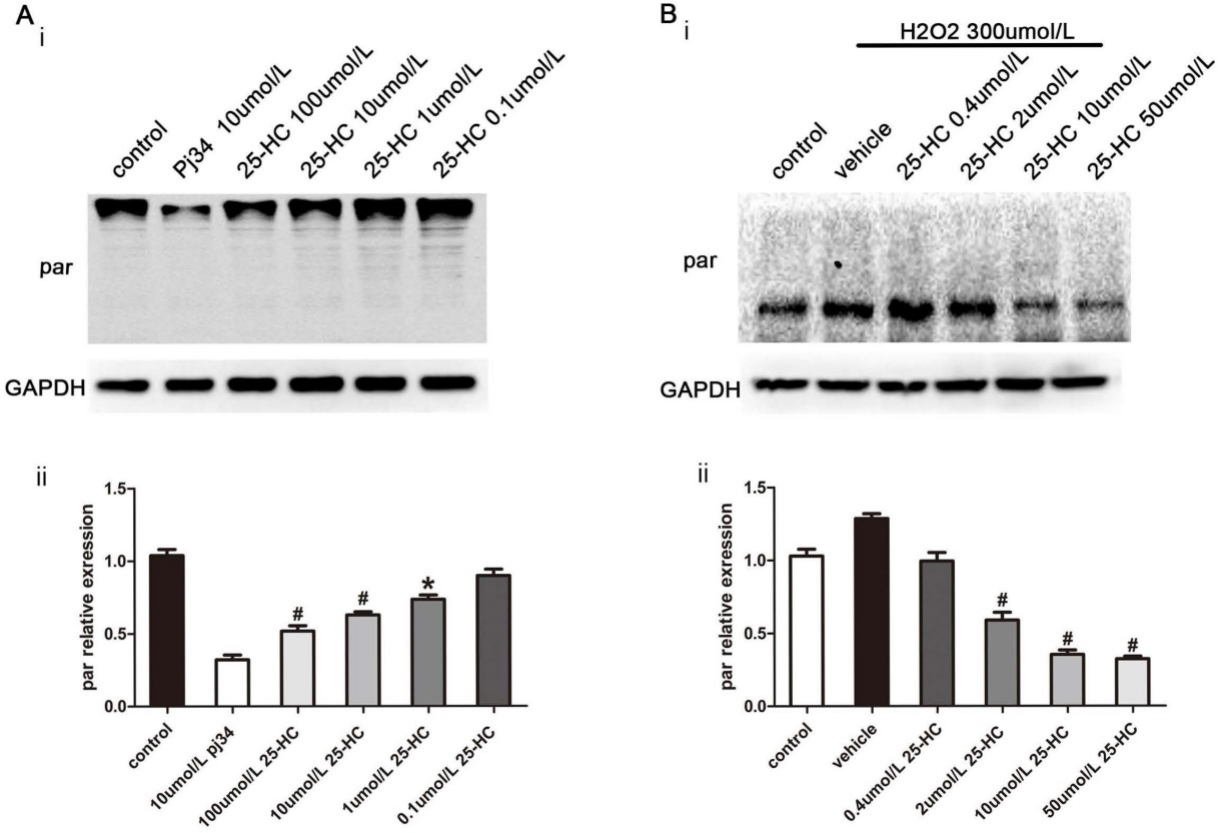
The PARP activity was inhibited by 25-HC *in vitro* and *in vivo*. (A) Nuclear extracts from primary mice myocardiocytes were incubated with different concentrations of 25-HC and then subjected to Western blot assay with anti-PAR antibody. (B) Neonatal C57BL/6 murine ventricular myocytes were subjected to H_2_O_2_ (300 μmol/L) stimulation to measure protein level of PAR.

**Figure 5:**
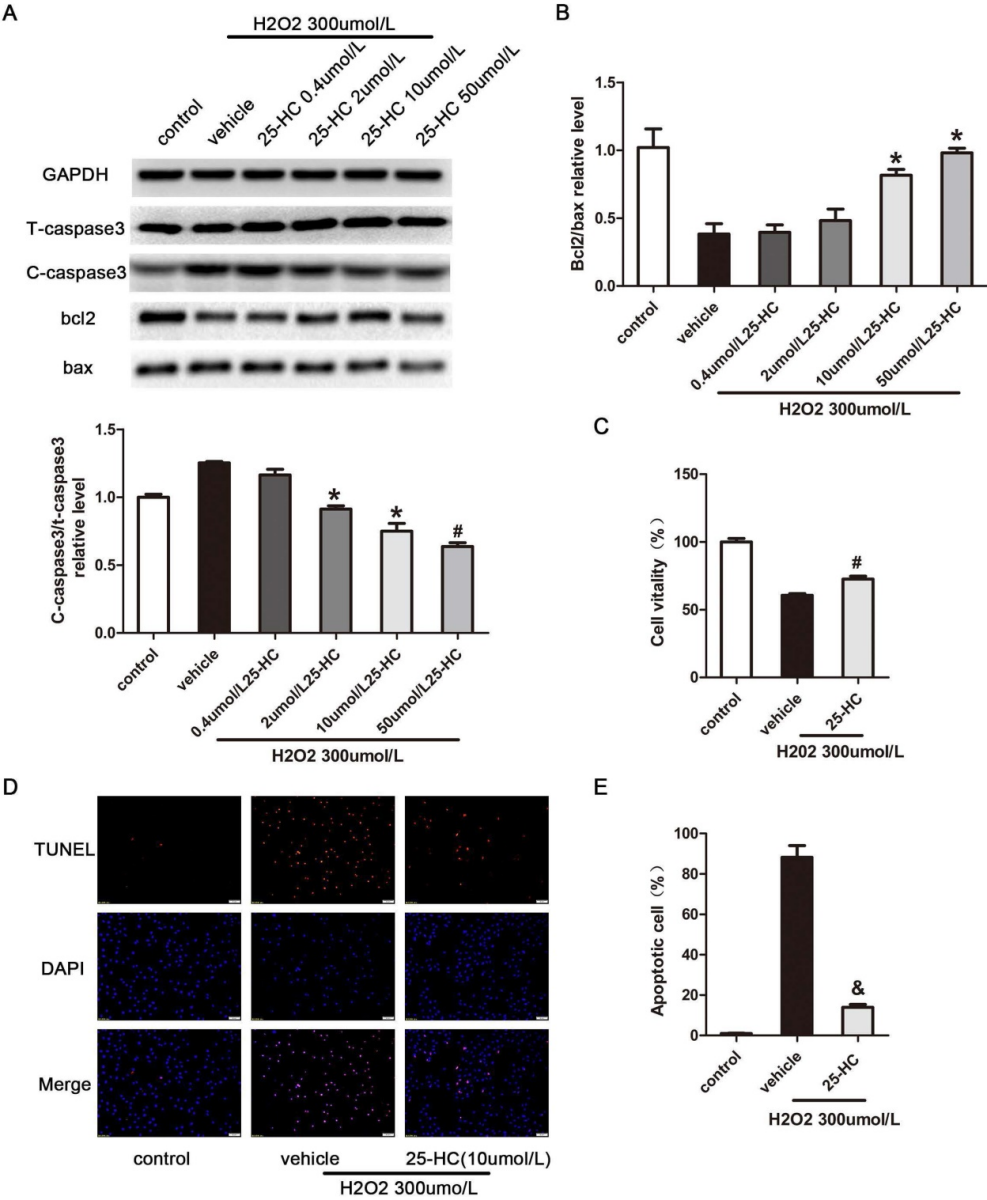
25-HC decreased H_2_O_2_-induced cell apoptosis. Neonatal C57/B6 myocardium was subjected to H_2_O_2_ (300 μmol/L) stimulation. (A) Myocardiocyte apoptosis-relative protein expression with representative gel blots; and (B) statistical results were illustrated. (C) Cell vitality was evaluated with the CCK8 assay. (D) Representative images of TUNEL staining (red) in sham and IR hearts. Nuclei were stained with 4,5-diamino-2-phenylindole (DAPI). Scale bar = 50 μm. (E) The number of TUNEL-positive apoptotic cardiomyocytes was expressed as the percentage of total nuclei. Mean ± SEM. N = 6, *P < 0.05 vs. vehicle group, #P < 0.01 vs. vehicle group, and P < 0.001 vs. vehicle group.

To confirm the relationship between 25-HC and PARP, nuclear extracts from primary mice cardiomyocytes were subjected to the PARP activity assay. The results showed that 25-HC inhibited the PARP activity in a dose-dependent manner *in vitro* (Figure 4A). Furthermore, primary mice cardiomyocytes were treated with hydrogen peroxide (H_2_O_2_), a widely accepted PARP1 activator 26. Consistent with previous findings, pre-incubation with 25-HC significantly inhibited the H_2_O_2_-induced expression of PAR-polymer proteins in dose dependent manner (Figure 4B). Furthermore, H_2_O_2_ significantly enhanced the expression of cleaved caspase-3 and decreased the Bcl-2/Bax ratio; conversely, these were significantly inhibited through the protective role of 25-HC in a dose-dependent manner (Figure 5A and 5B). Besides, our CCK-8 assay showed that the H_2_O_2_ treatment reduced the cellular viability of cardiomyocytes and that 25-HC could attenuate this effect (Figure 5C). Meanwhile, H_2_O_2_ treatment increased the number of TUNEL-positive cardiomyocytes, and 25-HC treatment notably down-regulated the apoptosis of cardiomyocytes (Figure 5D, and 5E). These findings suggested that 25-HC protected cardiomyocytes against IR injury by inhibiting the PARP activity.

### PARP1 knockout abolished the cardioprotective effect of 25-HC

**Figure 6:**
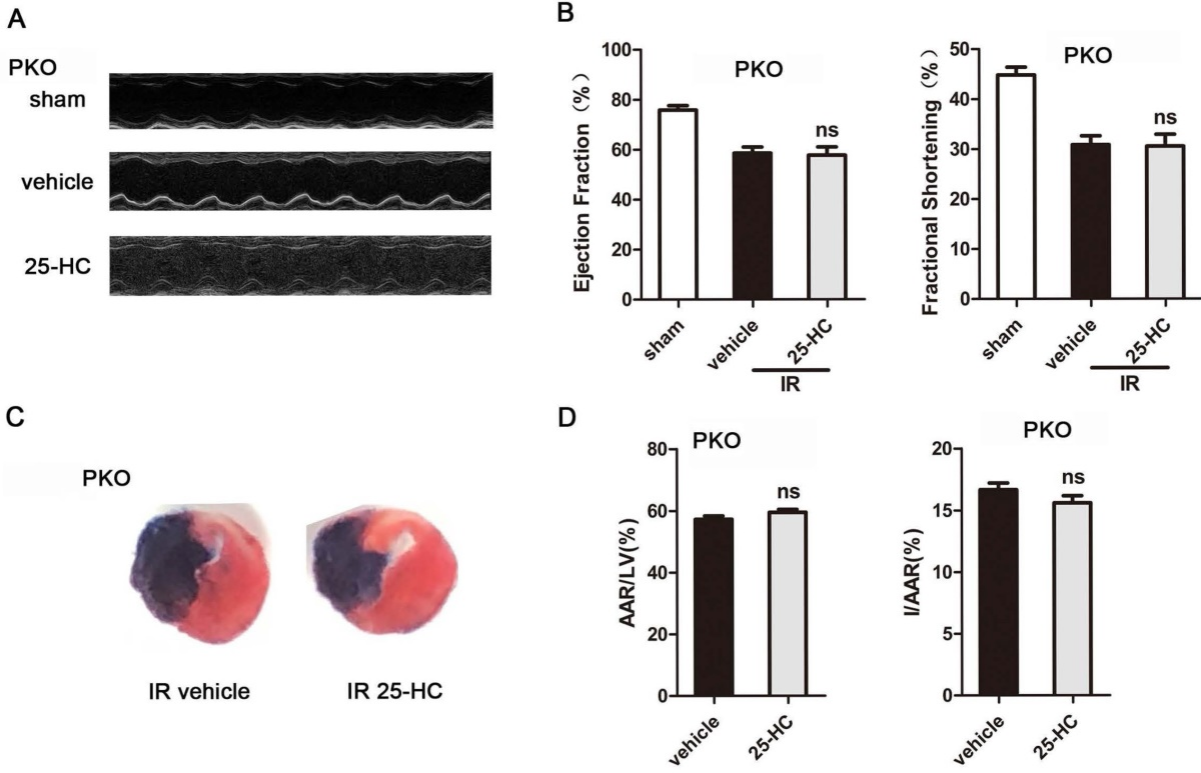
PARP1 knockout abolished the cardioprotective effect of 25-HC. Cardiac function post-IR in WT and PARP1 KO mice (A) Representative M-mode images and (B) statistical results (ejection fraction and fractional shorting) illustrated that cardiac systole function was ameliorated by 25-HC pretreatment (10 mg/kg). (C) Representative photographs of Evans blue/TTC double-stained murine heart slices obtained 24 h after IR injury. (D) Graphic representation of the LV infarct size and AAR: infarct size relative to AAR (I/AAR) and AAR relative to left ventricle (AAR/LV). Mean ± SEM. N = 5-6 per group, *P < 0.05 vs. vehicle group, #P < 0.01 vs. vehicle group.

Being a nuclear NAD^+^ ADP-ribosyltransferase, PARP1 accounts for about 90 percent of cellular PARP activity 27. To further verify the role of the PARP activity in the cardioprotective effect of 25-HC, IR mice model with PARP1 knockout were generated. The results revealed markedly increased LVEF% and LVFS% and decreased cardiac infarct size in PARP1 KO mice compared to WT mice in IR surgery (EF: 58.59% ± 0.85% vs. 45.70% ± 0.62%, P < 0.001; FS: 30.85% ± 0.62% vs. 22.36% ± 0.45%, P < 0.001; I/AAR: 16.66% ± 0.22% vs. 27.88% ± 0.41%, P < 0.001; Figures 6A, 6B, 6C, and 6D). However, the 25-HC group abrogated the increase of the LVFS% and LVEF% in IR mice (EF: 57.44% ± 1.1% vs. 58.59% ± 0.85%; FS: 30.55% ± 0.74% vs. 30.85% ± 0.62%; Figure 6B). Moreover, PARP1 knockout abolished the inhibitory effect of 25-HC on infarct area post-IR (I/AAR: 15.6% ± 0.23% vs. 16.66% ± 0.22%; Figure 6D).

**Figure 7:**
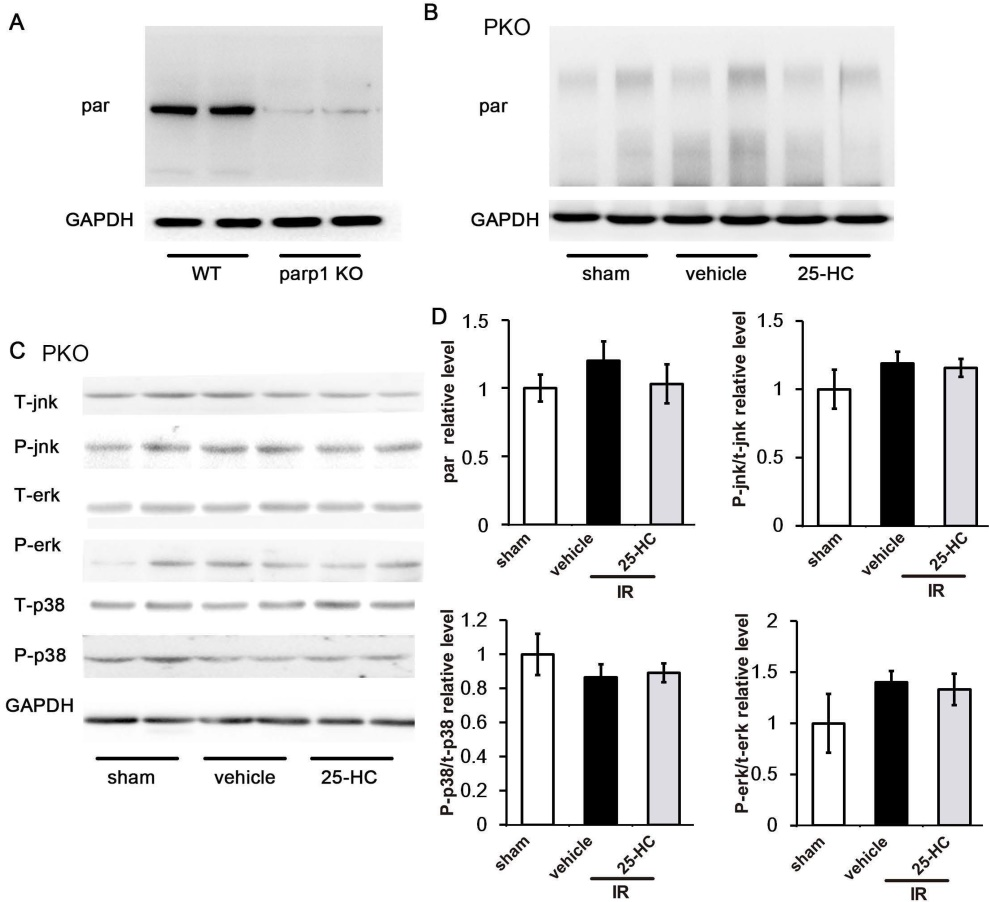
(A) and (B) PKO mice and C57/B6 mice were intraperitoneally injected with 25-HC in IR models, and the left ventricular tissues were subjected to Western blot assay with anti-PAR antibody. (C) PKO mice were intraperitoneally injected with 25-HC in IR models, and the left ventricular tissues were subjected to Western blot assay with anti-p38, JNK, and ERK antibodies. (D) Representative statistical results in each protein. Mean ± SEM. N = 5-6 per group.

To further explore the underlying mechanism, PKO mice and C57/B6 mice were intraperitoneally injected 25-HC in IR models. As expected, PARP1 knockout greatly reduced the PARP activity of IR heart (Figure 7A) and abrogated the inhibitory effect of 25-HC on the PARP activity assay (Figure 7B). These results suggested that PARP1 activity is essential for 25-HC to exert influence during IR injury. Besides, we also detected the MAPK signaling in IR injured PKO mice. Results revealed that phosphorylation of p38, JNK, and ERK was not significantly different between the vehicle group and 25-HC group in PKO mice (Figures 7C and 7D).

## Discussion

In the present study, we demonstrated that 25-HC inhibited IR-induced cardiac apoptosis. Furthermore, these protective effects are mediated via PARP activity inhibition. To our knowledge, this is the first study showing that this oxysterol reduces IR-induced cardiac apoptosis and exerts cardioprotective effects.

Instead of reducing ischemia-induced injury, reperfusion can even induce unpredictable reperfusion injury 2. Myocardial apoptosis is one of the critical pathogenic mechanisms underlying IR injury, and apoptosis indicates programmed cell death and removal without accompanying the activation of an inflammatory process in the light of DNA and cellular fragmentation 28. Apoptosis emerging within 24 h after IR injury would typically lead to declined cardiac contractile features and increased risk of the direct massive loss of myocytes, which can be major contributing factors to cardiac function decline 29. In addition, cardiomyocyte apoptosis also was triggered by H_2_O_2_ stimulation, which is responsible for activating apoptosis-related proteins 30. Meanwhile, PARP1 and MAPK signaling pathway were activated by H_2_O_2_ 25, 31. Consequently, we used H_2_O_2_ treatment to induce cardiomyocyte apoptosis *in vitro*, and the ROS-activated PARP-1 regulated the activation of MAPKs. Previous studies have also shown that the regulation was achieved by increased expression and enlarged cytoplasmic localization of MAPK phosphatase-1 (MKP1) upon PARP1 inhibition 26. Besides, PARP1 regulates the binding of ATF4/CREB2 with CRE elements that regulate MKP1 32. In our study, 25-HC treatment was cardioprotective against heart IR injury by anti-apoptosis through inhibiting PARP1 activation and phosphorylation of p38-MAPK and JNK1/2. Although our results verified the beneficial effect of 25-HC against acute IR injury, the long-term effects of 25-HC on cardiac remodeling and dysfunction of ischemic cardiomyopathy remain unclear. More researches are needed in the future in this regard.

Previous studies have proven that PARP1 participates in the progression of numerous cardiovascular diseases and the involved processes, such as apoptosis and autophagy^33^, ^34^. Furthermore, previous studies have proven that PARP inhibitors attenuated cardiac injury and apoptosis 10, 35. Furthermore, myocardial IR injury was ameliorated by genetically engineered PKO mice 33, 36, 37. These experiments with primary cardiomyocytes also proved that PARP is a protective molecule, which most likely acts by intercepting the apoptotic reaction of cardiomyocytes. In our study, we showed that 25-HC inhibited the PARP activity in *in vivo* and *in vitro* experiments. To our knowledge, this study is the first to report that 25-HC could protect against reperfusion injury-induced myocardial apoptosis by inhibiting the PARP activity. Although some studies have shown that 25-HC is associated with cell apoptosis 38, our study showed that increased 25-HC in the ischemic myocardium was associated with lesser PARP activation after IR due to the complex mechanisms involved. Moreover, our results also showed that 25-HC exerted a vital cardioprotective role against heart IR injury in WT mice. PARP1 KO mice ameliorated cardiac dysfunction and post-MI infraction area in IR surgery by suppressing PARP activation. Since PARP1 contributes to 90% of the total PARP activity 39, we also found that the anti-apoptosis effect of 25-HC was absent in PARP1 KO mice. These results demonstrated that PARP1 was necessary for 25-HC to protect myocytes.

Our experiment also showed that 25-HC could counterwork ventricular myocardial apoptosis by MAPK pathway. MAPK was a downstream factor in PARP- mediated cell apoptosis 24, 25, 40. The activation of MAPK is an essential component of cellular metabolism and plays a critical role in cardiomyocyte apoptosis during IR process 41. Besides, the pro-apoptotic effect of MAPK depends on the activity of caspase-3, JNK, and p38-MAPK signaling 42-44. Our results showed that the activation of p38-MAPK and JNK1/2 was significantly attenuated by the administration of 25-HC, which is consistent with previous reports 45, 46, whereas 25-HC also inhibited the expression of caspase-3 and apoptosis-related genes. Thus, we herein showed that 25-HC could participate in the process of myocardial ischemia injury by mediating the MAPK signal in cardiomyocytes.

In conclusion, our study presents 25-HC as a novel, potentially strategic agent for use against apoptosis in myocardial IR injury. 25-HC may represent a novel approach for the treatment of ischemic heart disease or myocardial ischemic reperfusion disease.

## Supplementary Material

Supplementary figure S1.

## Figures and Tables

**Table 1:** Sequences of PCR primers

Gene	Forward primers	Reverse primers
GAPDH	5'-CGTCCCGTAGACAAAATGGT-3'	5'-TTGATGGCAACAATCTCCAC-3'
TNFa	5'-AGGGTCTGGGCCATAGAACT-3'	5'-CCACCACGCTCTTCTGTCTAC-3'
IL-1b	5'GCAACTGTTCCTGAACTCAACT-3'	5'-ATCTTTTGGGGTCCGTCAACT-3'
IL-6	5'-TGGGGCTCTTCAAAAGCTCC-3'	5'-AGGAACTATCACCGGATCTTCAA-3'

## References

[1] Yellon DM, Hausenloy DJ (2007). Myocardial reperfusion injury. N Engl J Med.

[2] Braunwald E, Kloner RA (1985). Myocardial reperfusion: a double-edged sword?. J Clin Invest.

[3] Eefting F, Rensing B, Wigman J, Pannekoek WJ, Liu WM, Cramer MJ (2004). Role of apoptosis in reperfusion injury. Cardiovasc Res.

[4] MacLellan WR, Schneider MD (1997). Death by design. Programmed cell death in cardiovascular biology and disease. Circ Res.

[5] Gibson BA, Kraus WL (2012). New insights into the molecular and cellular functions of poly(ADP-ribose) and PARPs. Nature reviews Molecular cell biology.

[6] Schreiber V, Dantzer F, Ame JC, de Murcia G (2006). Poly(ADP-ribose): novel functions for an old molecule. Nature reviews Molecular cell biology.

[7] Hung TH, Skepper JN, Charnock-Jones DS, Burton GJ (2002). Hypoxia- reoxygenation: a potent inducer of apoptotic changes in the human placenta and possible etiological factor in preeclampsia. Circ Res.

[8] Fiorillo C, Ponziani V, Giannini L, Cecchi C, Celli A, Nassi N (2006). Protective effects of the PARP-1 inhibitor PJ34 in hypoxic-reoxygenated cardiomyoblasts. Cellular and molecular life sciences: CMLS.

[9] Rouleau M, Patel A, Hendzel MJ, Kaufmann SH, Poirier GG (2010). PARP inhibition: PARP1 and beyond. Nature reviews Cancer.

[10] Jagtap P, Szabo C (2005). Poly(ADP-ribose) polymerase and the therapeutic effects of its inhibitors. Nature reviews Drug discovery.

[11] Russell DW (2003). The enzymes, regulation, and genetics of bile acid synthesis. Annu Rev Biochem.

[12] Lin CY, Gustafsson JA (2015). Targeting liver X receptors in cancer therapeutics. Nature reviews Cancer.

[13] Hong C, Tontonoz P (2014). Liver X receptors in lipid metabolism: opportunities for drug discovery. Nature reviews Drug discovery.

[14] Schroepfer GJ Jr (2000). Oxysterols: modulators of cholesterol metabolism and other processes. Physiol Rev.

[15] Dang EV, McDonald JG, Russell DW, Cyster JG (2017). Oxysterol Restraint of Cholesterol Synthesis Prevents AIM2 Inflammasome Activation. Cell.

[16] Reboldi A, Dang EV, McDonald JG, Liang G, Russell DW, Cyster JG (2014). 25-Hydroxycholesterol suppresses interleukin-1-driven inflammation downstream of type I interferon. Science.

[17] Gold ES, Diercks AH, Podolsky I, Podyminogin RL, Askovich PS, Treuting PM (2014). 25-Hydroxycholesterol acts as an amplifier of inflammatory signaling. Proc Natl Acad Sci U S A.

[18] Goldstein JL, DeBose-Boyd RA, Brown MS (2006). Protein sensors for membrane sterols. Cell.

[19] McDonald JG, Russell DW (2010). Editorial: 25-Hydroxycholesterol: a new life in immunology. J Leukoc Biol.

[20] Schüle R, Siddique T, Deng H-X, Yang Y, Donkervoort S, Hansson M (2010). Marked accumulation of 27-hydroxycholesterol in SPG5 patients with hereditary spastic paresis. Journal of Lipid Research.

[21] Wencker D, Chandra M, Nguyen K, Miao W, Garantziotis S, Factor SM (2003). A mechanistic role for cardiac myocyte apoptosis in heart failure. The Journal of clinical investigation.

[22] Javadov S, Jang S, Agostini B (2014). Crosstalk between mitogen-activated protein kinases and mitochondria in cardiac diseases: therapeutic perspectives. Pharmacol Ther.

[23] Kyriakis JM, Avruch J (2012). Mammalian MAPK signal transduction pathways activated by stress and inflammation: a 10-year update. Physiol Rev.

[24] Hocsak E, Szabo V, Kalman N, Antus C, Cseh A, Sumegi K (2017). PARP inhibition protects mitochondria and reduces ROS production via PARP-1-ATF4-MKP-1-MAPK retrograde pathway. Free radical biology & medicine.

[25] Racz B, Hanto K, Tapodi A, Solti I, Kalman N, Jakus P (2010). Regulation of MKP-1 expression and MAPK activation by PARP-1 in oxidative stress: a new mechanism for the cytoplasmic effect of PARP-1 activation. Free radical biology & medicine.

[26] Racz B, Hanto K, Tapodi A, Solti I, Kalman N, Jakus P (2010). Regulation of MKP-1 expression and MAPK activation by PARP-1 in oxidative stress: a new mechanism for the cytoplasmic effect of PARP-1 activation. Free Radical Biology & Medicine.

[27] Kim MY, Mauro S, Gevry N, Lis JT, Kraus WL (2004). NAD+-dependent modulation of chromatin structure and transcription by nucleosome binding properties of PARP-1. Cell.

[28] Murphy E, Steenbergen C (2008). Mechanisms Underlying Acute Protection From Cardiac Ischemia-Reperfusion Injury. Physiological Reviews.

[29] Hausenloy DJ, Yellon DM (2008). Time to take myocardial reperfusion injury seriously. N Engl J Med.

[30] von Harsdorf R, Li P-F, Dietz R (1999). Signaling Pathways in Reactive Oxygen Species-Induced Cardiomyocyte Apoptosis. Circulation.

[31] Yu SW, Wang H, Poitras MF, Coombs C, Bowers WJ, Federoff HJ (2002). Mediation of poly(ADP-ribose) polymerase-1-dependent cell death by apoptosis-inducing factor. Science.

[32] Hocsak E, Szabo V, Kalman N, Antus C, Cseh A, Sumegi K (2017). PARP inhibition protects mitochondria and reduces ROS production via PARP-1-ATF4-MKP-1-MAPK retrograde pathway.

[33] Szabó C, Dawson VL (1998). Role of poly(ADP-ribose) synthetase in inflammation and ischaemia-reperfusion. Trends in Pharmacological Sciences.

[34] Pacher P, Szabo C (2007). Role of poly(ADP-ribose) polymerase 1 (PARP-1) in cardiovascular diseases: the therapeutic potential of PARP inhibitors. Cardiovascular drug reviews.

[35] Szabó C (2005). Cardioprotective effects of poly(ADP-ribose) polymerase inhibition. Pharmacological Research.

[36] Petrilli V, Herceg Z, Hassa PO, Patel NS, Di Paola R, Cortes U (2004). Noncleavable poly(ADP-ribose) polymerase-1 regulates the inflammation response in mice. J Clin Invest.

[37] Virag L, Szabo C (2002). The therapeutic potential of poly(ADP-ribose) polymerase inhibitors. Pharmacol Rev.

[38] Vurusaner B, Leonarduzzi G, Gamba P, Poli G, Basaga H (2016). Oxysterols and mechanisms of survival signaling. Mol Aspects Med.

[39] Chiarugi A, Moskowitz MA (2002). PARP-1-a Perpetrator of Apoptotic Cell Death?. Science.

[40] Osipov RM, Bianchi C, Feng J, Clements RT, Liu Y, Robich MP (2009). Effect of hypercholesterolemia on myocardial necrosis and apoptosis in the setting of ischemia-reperfusion. Circulation.

[41] Rose BA, Force T, Wang Y (2010). Mitogen-activated protein kinase signaling in the heart: angels versus demons in a heart-breaking tale. Physiol Rev.

[42] Ma XL, Kumar S, Gao F, Louden CS, Lopez BL, Christopher TA (1999). Inhibition of p38 mitogen-activated protein kinase decreases cardiomyocyte apoptosis and improves cardiac function after myocardial ischemia and reperfusion. Circulation.

[43] Borsello T, Clarke PG, Hirt L, Vercelli A, Repici M, Schorderet DF (2003). A peptide inhibitor of c-Jun N-terminal kinase protects against excitotoxicity and cerebral ischemia. Nat Med.

[44] Han BH, DeMattos RB, Dugan LL, Kim-Han JS, Brendza RP, Fryer JD (2001). Clusterin contributes to caspase-3-independent brain injury following neonatal hypoxia-ischemia. Nat Med.

[45] Milano G, Morel S, Bonny C, Samaja M, von Segesser LK, Nicod P (2007). A peptide inhibitor of c-Jun NH2-terminal kinase reduces myocardial ischemia-reperfusion injury and infarct size in vivo. American Journal of Physiology-Heart and Circulatory Physiology.

[46] Bassi R, Heads R, Marber MS, Clark JE (2008). Targeting p38-MAPK in the ischaemic heart: kill or cure?. Curr Opin Pharmacol.

