# Supplementary figures and images for: 25-Hydroxycholesterol protects against myocardial ischemia-reperfusion injury via inhibiting PARP activity
Source: Int J Biol Sci. 2020 Jan 1;16(2):298–308. doi: 10.7150/ijbs.35075 (PMC6949155; doi:10.7150/ijbs.35075)

S1

A

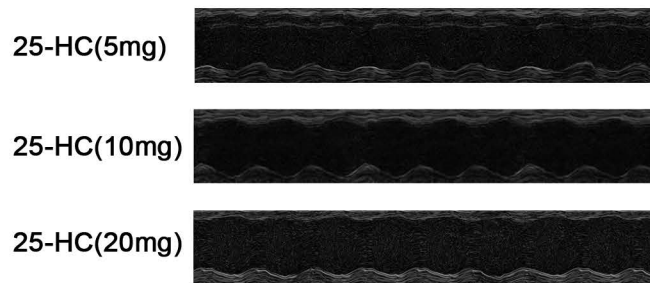

B

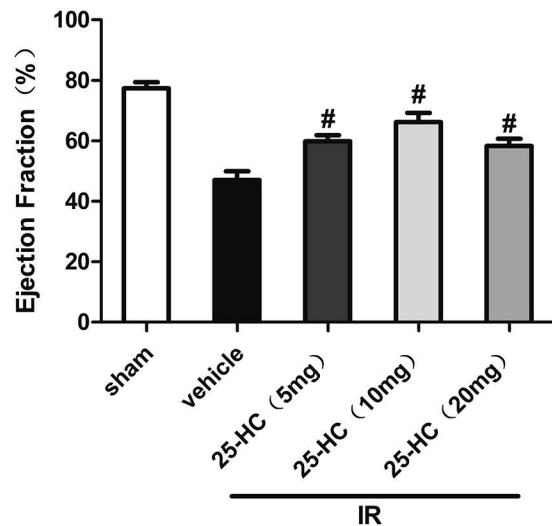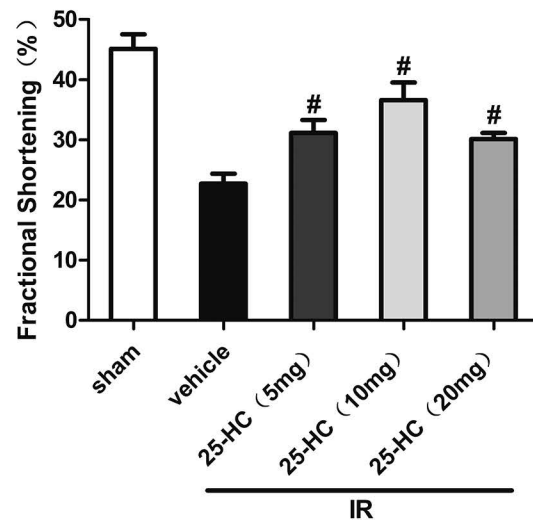

Supplement: Supplementary file 1 — Supplementary figure S1. [file ijbsv16p0298s1.pdf]
